# The First WHO International Standard for Harmonizing the Biological Activity of Bevacizumab

**DOI:** 10.3390/biom11111610

**Published:** 2021-10-30

**Authors:** Haiyan Jia, Parvathy Harikumar, Eleanor Atkinson, Peter Rigsby, Meenu Wadhwa

**Affiliations:** 1 Division of Biotherapeutics, National Institute for Biological Standards and Control, Hertfordshire EN6 3QG, UK; parvathy.harikumar@nibsc.org (P.H.); meenu.wadhwa@nibsc.org (M.W.); 2 Division of Technology Development and Infrastructure, National Institute for Biological Standards and Control, Hertfordshire EN6 3QG, UK; eleanor.atkinson@nibsc.org (E.A.); peter.rigsby@nibsc.org (P.R.)

**Keywords:** angiogenesis, Bevacizumab, bioassay, biosimilar, HUVEC, international standard, monoclonal antibody, oncology, ophthalmology, VEGF

## Abstract

Several Bevacizumab products are approved for clinical use, with many others in late-stage clinical development worldwide. To aid the harmonization of potency assessment across different Bevacizumab products, the first World Health Organization (WHO) International Standard (IS) for Bevacizumab has been developed. Two preparations of a Bevacizumab candidate and comparator were assessed for their ability to neutralize and bind vascular endothelial growth factor (VEGF) using different bioassays and binding assays in an international collaborative study. Relative potency estimates were similar across different assays for the comparator or the duplicate-coded candidate sample. Variability in relative potency estimates was reduced when the candidate standard was used for calculation compared with various in-house reference standards, enabling harmonization in bioactivity evaluations. The results demonstrated that the candidate standard is suitable to serve as an IS for Bevacizumab, with assigned unitages for VEGF neutralization and VEGF binding activity. This standard coded 18/210 was established by the WHO Expert Committee on Biological Standardization, which is intended to support the calibration of secondary standards for product development and lifecycle management. The availability of IS 18/210 will help facilitate the global harmonization of potency evaluation to ensure patient access to Bevacizumab products with consistent safety, quality and efficacy.

## 1. Introduction

Neovascular diseases, including cancers and intraocular diseases, are hallmarked by excess new blood vessels, which arise from pre-existing ones in the form of angiogenesis [1]. This process is driven by local tissue hypoxia due to an increasing metabolic demand to microcirculation in the case of neoplasm growth and progression. In eye disorders such as diabetic retinopathy and neovascular age-related macular degeneration, tissue hypoxia is mainly caused by either hyperglycemia or aging-induced capillary loss and ischemia. Tissue hypoxia in turn triggers overexpression of pro-angiogenic factors including vascular endothelial growth factor (VEGF) [2]. As a member of the VEGF family, VEGF-A exists in several different protein isoforms due to the alternative splicing of VEGF-A mRNA and the VEGF-A165 isoform (referred to hereafter as VEGF), and is the major pro-angiogenic factor secreted by many human tissues. VEGF exerts its biological activity mainly through binding to it signaling receptor, VEGF receptor 2 (VEGFR2), on the surface of vascular endothelial cells (ECs)—activating downstream signaling pathways for EC migration and proliferation and leading to angiogenesis. In neovascular disease, VEGF is dominantly upregulated and the expression level of VEGF correlates with neovascular density and metastatic spread in some cancer types, including colorectal, breast and cervical cancers. Consequently, VEGF is recognized as a key target for anti-angiogenic therapy [2,3]. Four structurally different VEGF antagonists have been approved by the US Food and Drug Administration (FDA) and the European Medicines Agency (EMA; Table 1). These anti-VEGF biotherapeutics have transformed the treatment of certain oncologic and ophthalmic diseases.

Bevacizumab, a monoclonal antibody (mAb) against VEGF, was the first anti-angiogenic biological drug to be developed [4,5]. The mechanism of action (MOA) for Bevacizumab is through binding with high affinity to soluble VEGF via its antigen-binding fragment (Fab) region, thereby sterically blocking the interaction of VEGF with VEGFR2 on ECs and subsequently interrupting downstream signaling for angiogenesis [1,2,3]. Thus, VEGF neutralisation by Bevacizumab regresses the neovascularisation of tumours and inhibits tumour growth, as well as suppressing pathological angiogenesis and vessel hyper-permeability in intraocular diseases. Originally approved for the first-line treatment of metastatic colorectal cancer [6,7,8,9], current therapeutic indications for Bevacizumab include metastatic colorectal cancer, metastatic breast cancer, non-small-cell lung cancer, glioblastoma, renal cell carcinoma, ovarian cancer and cervical cancer [10]. Therefore, Bevacizumab has been added to the World Health Organization (WHO) Model List of Essential Medicines for a basic health care system [11]. In addition, Bevacizumab is also used off-label to treat eye diseases including neovascular age-related macular degeneration and diabetic macular edema [12,13,14]. More recently, Bevacizumab is under investigation to control neovascularization-related pulmonary edema and to mitigate the life-threatening acute respiratory distress syndrome in patients with severe coronavirus disease 2019 [15,16], which may result in a potential extension of its clinical indication.

**Figure 1:**
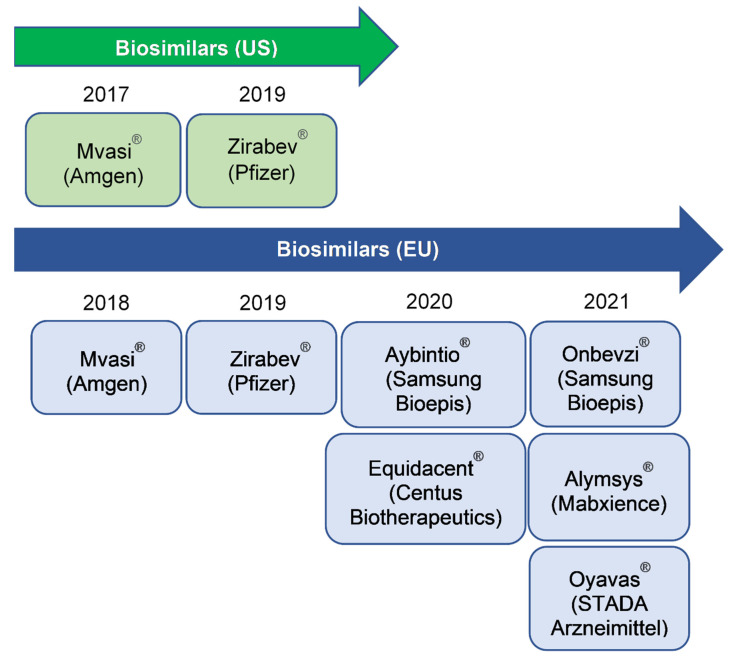
Biosimilars for Bevacizumab in US and EU, and their approval year.

The expiration of the patent protecting the originator product Avastin^®^, which achieved global sales of US$6.8–7.1 billion between 2017–2019, has motivated intense biosimilar development [17]. Since the approval of the first biosimilar, Mvasi^®^/Bevacizumab-AWWB (Amgen) by the US FDA in September 2017 and by the EMA in January 2018 for indications in these jurisdictions (except for ovarian cancer in the US due to orphan drug exclusivity) [18,19,20,21], a second biosimilar, Zirabev^®^/Bevacizumab-BVZR (Pfizer) was approved by these regulatory agencies (EMA February 2019; FDA June 2019) [22,23,24]. Both biosimilars for Bevacizumab were launched in the US in 2019. In the European Union, numerous biosimilars [25,26,27,28] have been sequentially authorised for use (Figure 1). A number of other potential biosimilars of Bevacizumab are currently in late-stage clinical development worldwide, with some anti-VEGF mAb products available in Argentina, India and Russia [29]. The availability of biosimilars offers the opportunity for increasing patient access to life- and vision-saving therapies across oncologic and ophthalmic indications and for more cost-efficient choices with potentially lower overall expenditures of healthcare systems [30].

By recombinant DNA technology, Bevacizumab is produced in Chinese hamster ovary (CHO) cells [4,5]. It is a full-length humanized IgG1κ mAb composed of two identical light chains (214 amino acid residues) and two heavy chains (453 amino acid residues) with a total molecular weight of 149 kDa. The heavy chains demonstrate C-terminal heterogeneity (lysine variants) and also contain one N-linked glycosylation site at asparagine 303. Like other therapeutic mAbs produced from living cells, Bevacizumab is a large, highly complex molecule and heterogeneous by nature. The structural and activity profiles of mAbs are influenced by many parameters, ranging from host cell lines and cell culture conditions to biosynthesis events including post-translational modifications (e.g., glycosylation) and downstream purification processes as well as during formulation and storage [31,32,33], and even small changes causing alteration of critical quality attributes can have significant implications for safety and efficacy. In addition, post-authorization changes commonly occur for mAbs, and this can be associated with a shift in the in vitro biological activities of the products—as noted with Rituximab and Trastuzumab [34,35]. Therefore, the quality profiles of biosimilar products should be monitored in terms of their structural and functional properties throughout the period of product development and production, and thereafter long-standing management of the product lifecycle.

In response to the increased development and availability of biosimilar products, there is a need for international standards (ISs) for VEGF antagonists to control assay performance and harmonize potency assessment. The WHO Expert Committee on Biological Standardization (ECBS) recognized this need and endorsed the initiation of IS development for these products in October 2016 [36]. To fulfil NIBSC’s mission in assuring the quality of biological medicines [37,38,39], we produced lyophilized Bevacizumab preparations, including a candidate standard, and organised an international collaborative study with the aims of (i) developing a WHO IS for Bevacizumab by assessing the suitability of the candidate preparation to serve as a “reference standard” for bioactivity determination of Bevacizumab products and (ii) assigning international units of activity to the different functionalities of the proposed Bevacizumab IS.

## 2. Materials and Methods

### 2.1. Materials and Processing

A batch of bulk drug substance of recombinant Bevacizumab was kindly donated to the WHO (see Acknowledgement). A suitable certificate of analysis was also provided. The drug product, Avastin^®^ (Roche) was purchased to serve as a comparator. Both candidate and comparator materials were expressed in CHO cells. Trial fills were conducted using two different formulations: (A) 25 mM sodium citrate tribasic dihydrate, 150 mM sodium chloride, 1% human serum albumin, pH 6.5 and (B) 10 mM *L*-histidine, 10 mM l-histidine hydrochloride monohydrate, 1% d-trehalose dihydrate, 0.01% polysorbate-20, 1% human serum albumin, pH 6.2. The biological activity of the lyophilized preparations was compared with the bulk material in cell proliferation inhibitory assays using primary human umbilical vein endothelial cells (HUVECs). Binding was examined using an immunoassay and a surface plasmon resonance (SPR)-based biosensor assay.

The definitive fill of the candidate standard (NIBSC code 18/210) and two small-scale fills of a comparator preparation and an additional sample (NIBSC codes 18/214 and 18/216) were carried out at NIBSC using WHO ECBS guidelines [40]. For this, buffers and excipients (25 mM Sodium citrate tribasic dihydrate, pH 6.5, 150 mM Sodium chloride, 1% Human serum albumin) were prepared using nonpyrogenic water and depyrogenated glassware and solutions filtered using sterile nonpyrogenic filters (0.22 µM Stericup filter system, Millipore, Burlington, MA, USA) where appropriate. For both preparations of candidate (18/210) and comparator (18/214), a solution of Bevacizumab at a theoretical protein concentration, given as ‘predicted µg’ (calculated from the dilution of the bulk material or the drug product Avastin^®^ of known protein mass content, as provided by the manufacturer), was distributed in 1 mL aliquots into 5 mL ampoules. The candidate preparation 18/210 (containing approximately 53 µg of Bevacizumab per ampoule) was coded in duplicate as A and C, while the comparator preparation 18/214 (containing approximately 50 µg of Bevacizumab per ampoule) was coded as B. A solution of candidate Bevacizumab (from the same bulk drug substance as used for 18/210) at a 20% lower theoretical protein content than the candidate preparation 18/210 was also dispensed in 1 mL aliquots to generate the preparation 18/216 (containing approximately 43 µg of Bevacizumab per ampoule) to serve as an additional sample with the code of D. All preparations were lyophilized under optimized and controlled conditions, and the glass ampoules were sealed under dry nitrogen by heat fusion and stored at −20 °C in the dark.

For each fill, a percentage of ampoules were weighed, and the residual moisture and headspace oxygen content of each preparation were measured by the coulometric Karl–Fischer titrator (Mitsubishi CA-200, supplied through A1-Enviroscinces Ltd., Blyth, UK)) and frequency modulated spectroscopy using the Lighthouse FMS-760 Instrument (Lighthouse Instruments Ltd., Charlottesville, USA), respectively. Testing for microbial contamination using the total viable count method did not show any evidence of microbial contamination.

### 2.2. Collaborative Study Participants

A total of twenty-five laboratories from eleven different countries, listed in Table 2, kindly participated in the study and contributed to the assay data analysed for the study. The participants included seven regulatory/control laboratories, one pharmacopeial laboratory, fourteen biopharmaceutical product manufacturers, two contract research organisations and one commercial vendor.

### 2.3. Collaborative Study Design

Participating laboratories were provided with a sample pack, which consisted of 5 ampoules each of the study samples A–C, for each assay type to be undertaken. Some laboratories were also sent sample D, which contained the same material as sample A but had a lower protein content than sample A, for assessing the sensitivity of the assays to detect differences. The 1st WHO Reference Reagent (RR) for VEGF165 (NIBSC code 02/286) was provided as a common preparation to participants performing VEGF165 neutralization assays to reduce assay variability arising from the use of different human VEGF165 preparations. A study protocol, which stated the study aims and instructions for reconstituting study samples and performing assays, along with spreadsheets for reporting raw data and assay details, was provided.

Participants were advised to (a) evaluate the study samples in a pilot assay for each of the assay types to ensure appropriate assay conditions and optimal dose–response curves prior to assay runs for the study, (b) select a suitable concentration of VEGF165 RR (02/286) for use in VEGF165 neutralization bioassays and (c) following the establishment of suitable assay conditions, test all samples concurrently at least on three separate occasions using their own routine methods, within a specified plate layout which allocated the samples across 3 or 4 plates and allowed testing of replicates. Participants were requested to test dilution series of samples A–C and their in-house reference standards on each plate using freshly reconstituted ampoules for each assay.

For binding assays, participants were requested to perform three independent assays on three separate occasions using proprietary assays or in-house established assays to assess the binding of study samples and their in-house reference standards to VEGF165 sourced from a commercial supplier.

### 2.4. Assays Employed in the Study

For VEGF165 neutralization (Table S1), three different bioassays reflecting the MOA of Bevacizumab were used [1]. These included (i) endothelial cell proliferation inhibitory assays based on Bevacizumab blockade of VEGF165-stimulated proliferation of primary human umbilical vein endothelial cells (HUVECs) [41,42,43,44], (ii) reporter gene assays (RGAs) using a stable human embryonic kidney (HEK293) cell line transfected with VEGFR2 and the VEGF165 responsive element nuclear factor of activated T cells (NFAT) upstream of a luciferase reporter gene [45,46], along with other VEGF-responsive ready-to-use cell line-based RGAs, and (iii) enzyme-fragment complementation (EFC) assays using HEK293 cell lines transfected with VEGFR2 and two β-galactosidase fragments through VEGFR2 dimerization, causing EFC-mediated activation of β-galactosidase [47,48].

Binding assay platforms included a direct enzyme-linked immunosorbent assay (ELISA) using immobilized VEGF165 and horseradish peroxidase (HRP)-conjugated antibody, a competitive immunoenzymetic assay using biotinylated anti-rhVEGF/avidin-HRP and a BioLayer interferometry (BLI) system using fiber-optic biosensors for detection (Table S2).

All study participants used their own qualified assays with their own assay acceptance criteria and in-house reference standards where available.

### 2.5. Stability Studies

Accelerated temperature degradation (ATD) studies were performed to predict the long-term stability of the candidate standard. Ampoules of the definitive fill of the candidate preparation (NIBSC code 18/210) were stored at a range of elevated temperatures (4 °C, 20 °C, 37 °C and 45 °C) and tested at indicated time points together with ampoules stored at the recommended temperature of −20 °C and −70 °C as a baseline reference temperature. Where possible, relative bioactivities of the ATD samples were used to fit an Arrhenius equation relating the degradation rate to absolute temperature, assuming first-order decay [49], and hence to predict the degradation rate when stored at −20 °C.

For stability assessment after reconstitution, samples of the candidate standard 18/210 were reconstituted and left at 4 °C or room temperature for either 1 day or 1 week. The reconstitutions were timed to allow all samples to be assayed concurrently against a freshly reconstituted ampoule. For freeze–thaw assays, samples of the candidate standard 18/210 were reconstituted and subjected to a series of freeze–thaw cycles (up to 4 cycles). They were then assayed concurrently with a freshly reconstituted ampoule.

### 2.6. Statistical Analysis

An independent centralized statistical analysis of all bioassay data was performed at NIBSC. Analysis of dose–response curve data was performed using a four-parameter logistic (sigmoid curve) model as defined by the following equation:

(1) $$y=\;\alpha \;-\frac{\delta }{1+10^{\beta \left(\log_{10}x-\log_{10}\gamma \right)}}$$

where y denotes the assay response, x is the concentration, α is the upper asymptote, δ is the difference between upper and lower asymptotes, β is the slope factor and γ is the EC_50_ (50% effective concentration). Assay responses were log transformed for neutralization (HUVEC/RGA/EFC) assays while no transformation of assay response was used for binding assays. Models were fitted using the R package ‘drc’ [50,51]. Parallelism (similarity) for a pair of dose–response curves was concluded by demonstrating equivalence of the parameters α, β and δ. Equivalence-bound values and the methods for determining them are described in the Results section. In three cases (laboratories 03, 07a and 07b) a parallel line model was used, and equivalence criteria applied to the β parameter in the sigmoid curve model analysis were used to confirm parallelism of the samples tested.

Where satisfactory parallelism was concluded for a sample, the model was fitted to both the sample and the standard, with common values of α, β and δ to directly estimate its relative potency. All relative potency estimates were combined to generate unweighted geometric mean (GM) potencies for each laboratory, and these laboratory means were used to calculate overall unweighted geometric mean potencies. Variability between assays and laboratories has been expressed using geometric coefficients of variation (GCV = {10^s^−1} × 100% where s is the standard deviation of the log_10_ transformed potencies).

To assess the inhibitory effect of Bevacizumab, EC_50_ estimates were derived for each laboratory performing VEGF neutralization assays. For the proposed IS, the inhibitory activity was determined by using the following equation:

Amount of Bevacizumab (IU) inhibiting a fixed amount of VEGF (Unit) = potency of preparation (IU) × EC_50_ (ng) Assumed mass content (ng)

where EC_50_ values are derived from HUVEC assays performed by selected laboratories using 25 Units of VEGF165 RR (NIBSC code 02/286), an assumed mass content for the Bevacizumab candidate standard (NIBSC code 18/210) is 50,000 ng and the proposed arbitrary unitage for the Bevacizumab candidate standard (NIBSC code 18/210) is 1000 IU.

## 3. Results

### 3.1. Evaluation of Bevacizumab Materials and Lyophilizing Formulations

**Figure 2:**
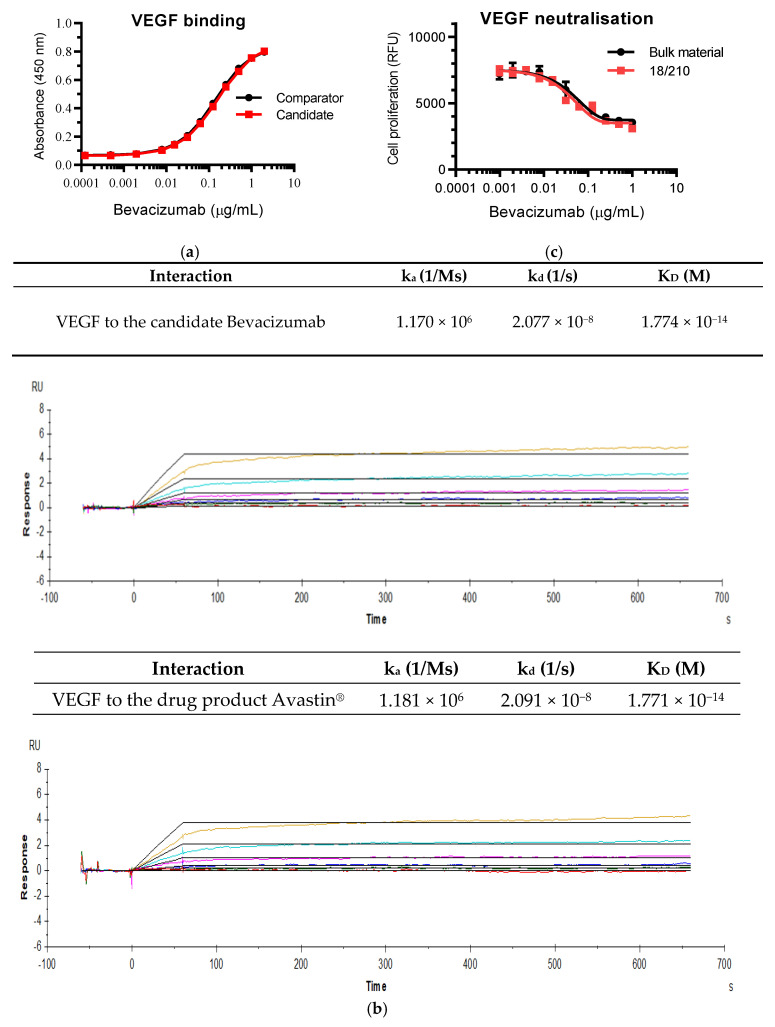
Binding activity of the candidate Bevacizumab bulk material and neutralising activity of the candidate preparation 18/210. (**a**) Direct binding by ELISA of the candidate Bevacizumab bulk material or the comparator Avastin^®^ to coated VEGF. Data for each point was represented as a mean and standard deviation (error bars) of three individual assay plates each containing samples in duplicate. (**b**) Surface plasmon resonance measurements of VEGF binding to captured Bevacizumab using a BIAcore T200 instrument (Cytiva, Uppsala, Sweden). Bevacizumab was captured by anti-human IgG-Fc antibodies immobilised on the chip surface, followed by sequential injections of VEGF (R&D Systems) at increasing concentrations over both the Bevacizumab-captured and the reference (non-Bevacizumab-captured) surfaces at a flow rate of 30 μL/min at 25 °C. The binding sensorgrams were double referenced prior to global fitting of the increasing concentrations of VEGF (colour coded ranging from 0.156 nM to 5 nM) as both blank running buffer (no VEGF) and blank surface (no Bevacizumab) were used as references for background subtraction. Association and dissociation rate constants (k_a_ and k_d_) were obtained by analysing and fitting data according to the 1:1 L binding model. Equilibrium affinity constant (K_D_) was derived from the kinetic parameters (K_D_ = k_d_/k_a_). (**c**) Inhibition of VEGF-stimulated cell proliferation by the candidate standard 18/210 in comparison with the bulk material. The VEGF neutralisation assays were performed in HUVEC-based bioassays. Data for each point was presented as a mean and standard deviation (error bars) of three individual assay plates, each containing samples in duplicate.

The candidate Bevacizumab bulk material was initially evaluated for its binding and neutralising activities in comparison with the drug product Avastin^®^ to assess its fitness of purpose prior to a pilot lyophilization. VEGF binding data showed the same dose–response curves with both materials (Figure 2a). Similarly, equal binding kinetics profiles (association and dissociation rate constants k_a_ and k_d_) and high binding affinities (K_D_ values) for VEGF165 were found between the two materials (Figure 2b). The biological activity of the candidate material was compared with the drug material in cell proliferation inhibitory assays using primary HUVECs and exhibited comparable potency for inhibition of VEGF165-dependent HUVEC proliferation (data not shown).

A trial fill was then conducted to test two different formulations for assessing the suitability of the formulation for the desired stability. The bioactivity of the lyophilized preparations was compared with the bulk material in HUVEC-based bioassays and binding assays. Although both formulations proved to be suitable, Formulation A was selected for the final lyophilization of the three preparations (Table 3) as this retained marginally more biological activity relative to the bulk material in comparison with Formulation B in both bioassays and binding assays (data not shown). Formulation A with sodium citrate and human serum albumin as excipients has been used previously for WHO ISs for mAbs, e.g., Infliximab IS and Adalimumab IS [38,39]. As shown in Table 3, all parameters of ampoule integrity were within the specifications required by the WHO for long-term stability. The potency of the candidate standard 18/210 was compared with the bulk starting material in HUVEC-based VEGF165 neutralization assays and showed very similar dose–response curves between them, suggesting that the candidate Bevacizumab material had been lyophilized successfully with no loss in bioactivity (Figure 2c).

### 3.2. Study Data Submitted by the Participants

The suitability of the lyophilized candidate Bevacizumab preparation 18/210 to serve as an IS for Bevacizumab bioactivity was evaluated in the international collaborative study. Participating laboratories have been anonymized by the assignment of laboratory code numbers, which were allocated randomly and were not representative of the order of listing in Table 2 to retain confidentiality in the study. Among twenty-five participants, twenty-three laboratories performed VEGF neutralization assays and fourteen laboratories performed VEGF binding assays listed in Table 4 and Table 5.

All participants examined the biological activities of three preparations, i.e., duplicates of the candidate IS 18/210 (Study codes A and C) and the comparator sample B. The additional sample D containing the same material as sample A, with approximately 20% less Bevacizumab content than sample A, was tested in some laboratories. The majority of laboratories used their proprietary in-house reference standards representing therapeutic Bevacizumab, while the rest of the participants used Avastin^®^, except for one laboratory using a research grade Bevacizumab. All participants provided raw data from the assays so a global analysis of assay validity could be applied to allow data to be treated equally and to allow data from different laboratories to be compared to each other.

### 3.3. Study Assay Validity

Equivalence bounds for each model parameter (α, β and δ) were determined separately for neutralization (HUVEC/RGA/EFC) assays and binding assays. These bounds were set using data returned for coded duplicate samples A and C from all laboratories. As the model parameters are expected to be equivalent when testing the same sample against itself, absolute differences in α, log_10_β and δ parameters for samples A and C were calculated for each plate and upper equivalence bounds set as the 95th percentile of these values, using all values obtained across the study. For neutralization assays this gave upper bounds of 0.06, 0.19 and 0.34 for the absolute difference in α, log_10_β and δ parameters, respectively. The upper bound for log_10_β corresponds to a slope factor ratio of 1.55. For binding assays this gave upper bounds 0.19, 0.11 and 0.20 for the absolute difference in α, log_10_β and δ parameters, respectively, and the upper bound for log_10_β corresponded to a slope factor ratio of 1.29. For two dose–response curves to be concluded as parallel, equivalence had to be demonstrated for all three parameters (α, β and δ). It should be noted that the equivalence bounds were intended for use in the analysis of data from this study only, in order to apply consistent criteria to all laboratories and assess their relative performance. The bounds should not be interpreted as suitable values for routine use in the assessment of assay validity within the collaborating laboratories and may be overly stringent or lenient in individual cases.

In several laboratories (6/29 cases for neutralization assays and 10/14 cases for binding assays) no invalid assays were noted, and in several others (16/29 cases for neutralization assays and 13/14 cases for binding assays), total invalidity rates were ≤ 25%. Although the cause of invalidity could be attributed to a particular curve parameter in some cases (e.g., laboratory 25b HUVEC assays showed a lack of similarity in slope factor for samples B and D compared to samples A and C), no general trend across the whole study was observed and levels of curve similarity were broadly similar across all the samples tested, including local in-house standards.

### 3.4. Potency Estimates Relative to the Candidate Standard 18/210

As per the study design, the candidate preparation 18/210 was coded in duplicate as samples A and C, whereas the comparator preparation was coded as sample B. In addition, sample D was included to test assay sensitivity. Using the candidate preparation sample A as a reference standard, the bioactivities of three preparations-sample B, sample C (coded duplicate) and sample D were assessed by the participating laboratories, which employed their own qualified assay approaches. Geometric mean (GM) potency estimates and associated geometric coefficient of variation (GCV) values for the study samples B, C and D calculated relative to the candidate standard sample A are shown in Table 6 and Table 7 for each individual laboratory performing VEGF165 neutralization assays using HUVEC assays and RGA/EFC assays, respectively, and in Table 8 for VEGF165 binding assays.

The primary HUVEC assays showed that GM potency estimates for samples B and C relative to sample A from individual laboratories ranged from 0.66 to 1.19 and 0.77 to 1.13, respectively (Table 6). The median intra-laboratory GCV value for these potency estimates was 10% (ranging from 3.17% to 124.2%). In the cell line-based RGAs and EFC assays, GM potency estimates ranged from 0.73 to 0.98 and 0.87 to 1.06 for samples B and C, respectively, with the median intra-laboratory GCV value of 12% ranging from 5.74% to 52.00% (Table 7). The levels of median intra-laboratory GCV value for all VEGF165 neutralization assays (laboratory 4 was excluded due to high intra-laboratory assay variability) were 11.22% ranging from 0.47% to 56.01% with an upper quartile of 19.14% (Table 6 and Table 7). For binding assays, GM potency estimates for samples B and C relative to sample A ranged from 0.77 to 0.99 and 0.76 to 1.15, respectively (Table 8). As expected, the median intra-laboratory GCV value for binding assays was as low as 8.06%, ranging from 2.10% to 31.92% with an upper quartile of 12.35%, indicating good intermediate precision in the participating laboratories (Table 8).

**Figure 3:**
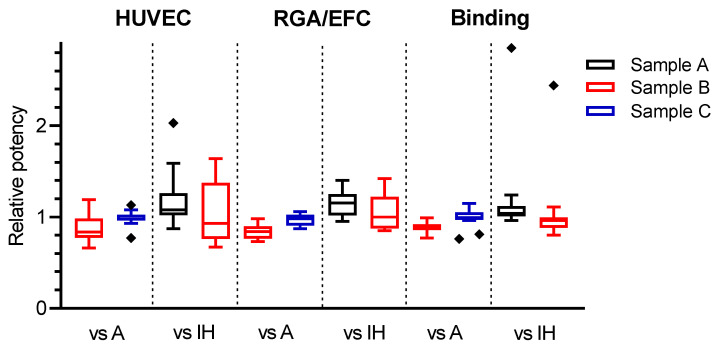
Laboratory geometric mean relative potency estimates calculated using either the candidate standard sample A (A) or individual in-house (IH) reference standards for VEGF neutralizing (HUVEC and RGA/EFC) and binding assays. Boxes represent the interquartile range and lines show the median. Diamonds indicate outliers.

In order to evaluate the overall combined potency estimates, some laboratories were excluded from further analysis. These included laboratories 25b (HUVEC assay) and 16 (binding assay), as both gave outlier results of < 0.80 for the relative potency of coded duplicate samples A and C (Figure 3). Furthermore, all laboratory GM estimates based on fewer than three valid individual assay estimates were also excluded. An overall summary of relative potency estimates following these exclusions is shown in Table 9. The overall potency estimates showed good agreement among different assay methods, with GM potency estimates of 0.86, 0.85 and 0.88, respectively, determined for sample B relative to sample A in HUVEC, RGA/EFC and binding assays (Table 9). The GM relative potency estimates for sample C were 1.01, 0.98 and 1.02 in HUVEC, RGA and binding assays, respectively, suggesting good agreement with the expected value of 1.00, as sample C is a coded duplicate of the candidate standard sample A. Collectively, these results showed that similar potency estimates for sample B or sample C relative to the candidate standard sample A were observed regardless of assay type used, i.e., across HUVEC assays, RGA/EFC and binding assays. Additionally, we investigated the lower potency estimates for sample B relative to sample A. The protein content of the candidate drug substance (sample A) and the comparator drug product (sample B) was quantified using the same instrument, method and extinction coefficient to minimise any differences due to different quantification approaches. We found that the estimated protein content of sample B was 87% of sample A. Therefore, we believe that the difference in relative potency between samples A and B in the study is most likely due to the difference in the protein contents between these two samples.

To assess the assay’s sensitivity to detecting differences, the biological activities of the additional sample D (the same material as sample A with approximately 20% lower protein content) were compared with sample A. Among various assays used by the laboratories to test sample D, the binding assays showed the lowest GM potency estimate relative to sample A of 0.81 (Table 9), indicating the consistency with 20% lower protein content. Interestingly, the neutralising activities of sample D relative to sample A in HUVEC assays and RGA/EFC assays revealed GM relative potency values of 0.84 and 0.88, respectively, suggesting that the HUVEC assay is slightly superior to the RGA/EFC assays for detecting differences. Nevertheless, these data showed that binding assays were more capable or adequately sensitive than neutralisation assays at detecting lower activity resulting from reduced protein content.

### 3.5. Improvement of Inter-Laboratory Variability by Use of the Candidate Standard 18/210

Potency estimates and associated GCV values for the study samples were also calculated relative to individual proprietary in-house reference standards where available for each laboratory, and are shown in Table 6, Table 7 and Table 8 for VEGF165 neutralization assays and binding assays. In-house reference standards used in the study are defined as those manufactured in-house for routinely supporting pre-clinical studies and product development by the participating manufacturers. Several other participants, including regulatory laboratories, used a clinical batch of the drug product Avastin^®^ or a research grade of anti-VEGF mAb, where an in-house reference standard was unavailable. Since laboratory 4 showed high intra-laboratory assay variability for the relative potency to in-house standards in the HUVEC assay (Table 6) and laboratory 19 revealed outliers (Figure 3) for the relative potency to in-house standards in the binding assay, they were both excluded from further analysis of the combined potency estimates.

As shown in Table 9, neutralization assays showed a greater level of inter-laboratory variability, giving the median GCV values of 27.98% and 30.29% for samples B and D relative to individual in-house standards, reflecting the wide distribution of bioactivity potency estimates determined for individual laboratories. However, when sample A was used for the calculation, the median GCV values decreased to 11.35% and 12.59%, respectively, for samples B and D (Table 9), which reflects a narrower distribution of potency estimates—suggesting an improved inter-laboratory variability by use of the common standard sample A (18/210). Similarly, the median inter-laboratory GCV values for samples B and D relative to in-house standards were observed to be relatively higher, at 9.61% and 11.80%, than those of 6.26% and 5.16% in Sample A in binding assays.

### 3.6. Estimates of EC_50_ Derived from Neutralisation Assays

To assess the inhibitory effect of the Bevacizumab preparation, laboratory GM EC_50_ estimates based on the assumed content of 50 µg for the samples together with the VEGF165 WHO RR (NIBSC code 02/286) concentration used by the laboratories in their VEGF neutralization assays were determined. There was a weak correlation between the EC_50_ value and the concentration of VEGF165 used by participants (Figure S1) and differing intercepts for fitted regression lines in HUVEC assays and RGA/EFC assays suggested that different dilutions of the candidate standard would be needed to neutralize a fixed amount of VEGF165 depending on the assay type (HUVEC or RGA/EFC). The poor correlation was due to a high variability between laboratories.

As shown in Table 10, EC_50_ estimates for Bevacizumab samples in HUVEC assays were determined from six laboratories using the same fixed VEGF165 WHO RR (NIBSC code 02/286) concentration of 25 Units/mL. With the proposed arbitrary unitage of 1,000 IU for Bevacizumab candidate IS (NIBSC code 18/210), the inhibitory activity was determined by taking the mean EC_50_ values of samples A and C (coded duplicate) derived for the HUVEC assays and by using the equation described in the Materials and Methods section. Based on this, 2.7 IU of Bevacizumab candidate IS (NIBSC code 18/210) inhibit the proliferative effect of 25 Units of VEGF165 WHO RR (NIBSC code 02/286) in the HUVEC assays.

### 3.7. Stability of the Candidate Preparations

To predict the long-term stability of the candidate standard and calculate an expected annual loss of bioactivity, an ATD study was carried out. Ampoules of Bevacizumab preparation (NIBSC code 18/210) stored at elevated temperatures (4 °C, 20 °C, 37 °C and 45 °C) up to 11 months after the definitive lyophilization were tested in comparison with ampoules stored at the recommended temperature of −20 °C and the baseline reference temperature of −70 °C in HUVEC assays. The potencies of all samples were expressed relative to the appropriate −70 °C baseline samples and the results showed that there was no detectable loss of bioactivity even at higher temperatures (Table S3). Since no loss in activity was evident following storage at any of the elevated temperatures, no predicted loss in bioactivity can be calculated at the current time, but data from ongoing ATD studies are expected to inform on the long-term stability of the IS. Further stability assessment of the candidate standard post reconstitution using HUVEC assays showed that the potency was retained after a week of storage of reconstituted samples at either 4 °C or room temperature (Table S4) or after four cycles of repeated freeze–thaw cycles of reconstituted samples (Table S5).

## 4. Discussion

In the present multi-centre collaborative study, the suitability of a lyophilised preparation of Bevacizumab candidate standard (NIBSC code 18/210) was assessed in biological assays and binding assays by the participating laboratories. The participants used their own qualified assays, which are routinely used to test Bevacizumab products, reflecting the MOA of Bevacizumab. This study has demonstrated that the candidate standard 18/210 could be used to obtain similar relative potency estimates for various samples across a range of different assay types performed by the participating laboratories. Importantly, the use of the candidate standard 18/210 to serve as a common reference standard for the calculation of the relative potency of samples reduced the inter-laboratory variability to enable a close agreement between the participating laboratories for each of the Bevacizumab bioactivities evaluated. In contrast, a poor agreement between the laboratories was found when relative potency estimates for the study samples were calculated by individual in-house reference standards, most likely reflecting the differences in individual proprietary in-house reference standards and a general lack of harmonization in bioactivity evaluation.

The potency assessment of Bevacizumab bioactivity in this study included its neutralization of VEGF-stimulated proliferation of primary HUVECs and VEGF-induced response from responsive cell lines based on its MOA. HUVECs are a well-established and often used cellular model for studying VEGF endothelial functions [41,42,43,44,52]. Besides expressing VEGFR2, HUVECs also express VEGFR1 as well as the co-receptors, neuropilins [1]. However, VEGFR2 is the key receptor primarily for VEGF signalling. VEGF binds to VEGFR2 on the EC surface and causes receptor dimerization and phosphorylation to elicit the activation of downstream signalling pathways resulting in cell proliferation. Therefore, the HUVEC proliferation inhibitory assay is a conventional quantitative bioassay, which measures the dose-dependent VEGF-neutralising activity of Bevacizumab following cell treatment. Although the HUVEC assay based on physiological relevance is a current method widely used by manufacturers for batch release testing, it is time-consuming (assay period of 4–5 days) and subject to a high degree of assay variability. Therefore, the use of immortalized cell line-based bioassays such as RGA and EFC assays has recently increased, and it is an alternative or preferred option. Such assays recapitulate the MOA of Bevacizumab, i.e., the blockade of VEGF–VEGFR2 binding mediated downstream of targeted gene expression of luciferase [45,46] or VEGFR2 dimerization-linked EFC, resulting in activation of β-galactosidase [47,48]. Ready-to-use cell lines for RGAs and EFC assays are now commercially available from several sources (e.g., Svar, Promega and DiscoverX). In contrast to HUVEC-based distal readouts of cell proliferation, these cell line-based VEGF neutralisation assays allow rapid readout with the assay period of 18–24 h and are expected to improve assay performance.

In this collaborative study, a majority of participating laboratories have performed HUVEC proliferation inhibitory assays for potency evaluation of Bevacizumab samples. The primary HUVECs used by the participants were sourced from a broad range of suppliers (e.g., Lonza, TCS Biologicals, Gibco, Thermo Fisher Scientific, ScienCell Research Laboratories and PromoCell). In terms of the VEGF-responsive cell line-based bioassays, 14 independent HEK293-based assays consisting of 12 RGAs and 2 EFC assays were conducted. We found that the level of intra-laboratory variability in the primary HUVEC-based assays was unsurprisingly high; however, it was not reduced in the cell line-based RGA/EFC bioassays with the combined overall VEGF neutralization assays, giving a median intra-laboratory GCV value of 11.22%. Since primary ECs are isolated from human umbilical veins, the assay variability of HUVEC-based VEGF neutralisation is mainly due to differences in the source, donors and cell passage numbers, although other factors are also likely to contribute. Nevertheless, similar GM relative potency estimates for samples B and C were observed between HUVEC assays and RGA/EFC assays.

The targeted VEGF binding of Bevacizumab through its Fab domain is the important step in preventing the interaction of VEGF with VEGFR2 upstream of the VEGF signalling pathway. Direct VEGF binding assays, which are fast and easily quantifiable, and provide a more sensitive way to detect differences in activity than VEGF neutralisation assays, were also included. Although Bevacizumab is able to bind Fcγ receptors and complement protein C1q through its fragment crystallizable (Fc) domain, it has been shown to lack antibody-dependent cell-mediated cytotoxicity and complement-dependent cytotoxicity activities [42,53]. Given that its Fab-mediated binding to VEGF is critical to the MOA of Bevacizumab, a total of 14 independent VEGF binding assays were carried out by the laboratories. As expected, the levels of intra-laboratory variability in binding assays were generally lower (GCV value: 8.06%) than those in cell-based VEGF neutralization assays.

The important finding of this study was the good agreement in Bevacizumab potency estimates when the candidate preparation sample A (NIBSC code 18/210) was used as a common standard, despite distinct methodologies employed by the participating laboratories. Data analysis of VEGF165 neutralization and binding assays revealed that the inter-laboratory GCV values for potency estimates of samples B–D relative to the candidate standard sample A were lower than those when various individual in-house reference standards were used for the calculation, which suggested the candidate standard 18/210 consistently improved inter-laboratory variability in different assays—highlighting the benefit of using the common standard sample A in comparison with individual in-house reference standards. The high variability associated with the combined GM potency estimates relative to in-house reference standards demonstrated a poor agreement between laboratories. This is because those proprietary in-house reference standards were established in individual laboratories from their own batches of Bevacizumab products based on their own manufacturing processes, which are unlikely to be comparable among different laboratories and therefore are unable to serve as a common standard. In contrast, the use of the candidate standard 18/210 as a common reference standard clearly showed increased harmonization of potency estimates among the participating laboratories. Indeed, this is the advantage of using the highest order public standard, i.e., a WHO IS for Bevacizumab bioactivity to minimize assay variability arising from using individual in-house reference standards from different laboratories.

ATD studies after 11-month storage of the candidate ampoules of 18/210 at elevated temperatures revealed no loss in bioactivity, indicating that the candidate standard 18/210 is sufficiently stable and will maintain long-term stability. Importantly, studies on stability monitoring of the candidate standard 18/210 will continue over the next few years to assess stability and any predicted loss in bioactivity over time. Further stability studies of the candidate standard 18/210 undertaken post-reconstitution have shown that potency is not reduced after 1 week of storage at either 4 °C or room temperature, or after 4 cycles of repeated freeze and thaw.

## 5. Conclusions

Taken together, these study results—including stability assessment—support the conclusion that the Bevacizumab preparation (sample A coded 18/210) is suitable to serve as the 1st IS for determining the in vitro bioactivity of Bevacizumab products. It was therefore established by the WHO Expert Committee on Biological Standardization in October 2020 as the 1st WHO IS for Bevacizumab, with assigned values of 1000 IU per ampoule for VEGF165 neutralizing activity and 1000 IU per ampoule for VEGF165 binding activity. This IS will help in controlling bioassay performance, support calibration of secondary or local standards and facilitate global harmonization and consistency in potency assessment across different Bevacizumab products. It should be noted that the IS is not intended for defining specific activity or for use as the reference product for biosimilarity determination. Furthermore, it is not intended for revising product labelling or changing the therapeutic dosage. It has to be emphasised that a clear distinction exists between WHO ISs and the reference products, since they serve different purposes and cannot be used interchangeably. The key difference in their roles reflects the fact that the reference products are used for all the comparability studies (i.e., biosimilarity), whereas WHO ISs are intended for use in calibrating assays, and cannot be used as reference products [54]. Importantly, the availability of this IS for determining in vitro bioactivity of Bevacizumab would ultimately support manufacturers at various stages of product development and lifecycle management, as well as in post-marketing surveillance, ensuring patient access to Bevacizumab products with consistency in safety, quality and efficacy.

## Figures and Tables

**Table 1:** Approved anti-VEGF biological medicines in the US and EU.

INN	Brand Name	Manufacturer	Product Type	US Approval	EU Approval
Bevacizumab	Avastin^®^	Genentech/Roche	Full antibody	2004	2005
Ranibizumab	Lucentis^®^	Genentech/Novartis	Fab fragment	2006	2007
Aflibercept	Eylea^®^	Regeneron	VEGFR1/2-Fc fusion protein	2011	2012
Brolucizumab	Beovu^®^	Novartis	Single-chain antibody fragment	2019	2020

INN: International non-proprietary name.

**Table 2:** Participants of the collaborative study.

Participants	Laboratory Address	Country
Akiko Ishii-Watabe and Takuo Suzuki	National Institute of Health Sciences, Division of Biological Chemistry and Biologicals, 3-25-26, Tonomachi, Kawasaki-ku, Kawasaki, Kanagawa, 210-9501	Japan
Chunping Deng	Bio-Thera Solutions Ltd., Bldg A6-5fl, 11 Kai-Yuan Blvd, Science City, Guangzhou, 510530	China
Feng Zhang and Lan Wang	National Institutes for Food and Drug Control (NIFDC), Division of Monoclonal Antibodies, No. 31 Huatuo Road, Daxing District, Beijing, 102629	China
Francesca Luciani and Agnese D’Angiò	ISS, Biologicals and Biotechnologicals Unit, National Centre for the Control and Evaluation of Medicines (CNCF), Istituto Superiore di Sanità, Viale Regina Elena 299, Rome, 161	Italy
Guoping Wu and Christian Erickson	R&D Systems, Bio-Techne, Bioassay, 614 McKinley Place NE, Minneapolis, MN55413	USA
He Chen and Jiemin Chen	Genor BioPharma, Building No. 3, 1690 Zhangheng Rd, Zhangjiang, Pudong District, Shanghai, 201203	China
Hongyan Ye and Jiulin Wang	Qilu Pharmaceutical, No. 243 Gong Ye Bei Road, Licheng District, Jinan, 250000	China
Jane Lamerdin and Ai Shih	Eurofins DiscoverX, 42501 Albrae Street, Fremont, CA94538	USA
Jianying Fu and Chen Ma	Henlius Biopharmaceuticals, 1289 Yishan Road, Shanghai, 200030	China
Jill Crouse-Zeineddini and Jolene Teraoka	Amgen Inc., One Amgen Center Dr., B30E Dropzone DZ-1B, Thousand Oaks, CA 91320	USA
Jixiang Jiao and Karen Zhang	Shanghai Roche Pharmaceuticals Ltd., 1100 Long Dong Avenue, Pudong District, Shanghai, 201203	China
Junxian Guo and Qingcheng Guo	Shanghai Biomabs Pharmaceuticals Co.,Ltd, NO. 301 Libing Road, Pilot Free Trade Zone, Shanghai, 201203	China
Karin Blume and Kerstin Mårtensson	Svar Life Science, Lundavägen 151, Malmö, 21224	Sweden
Keith Mortimer and Anita Carscadden	Therapeutic Goods Administration, TGA Laboratories, Biochemistry Section, 136 Narrabundah Lane, Symonston, Canberra ACT, 2609	Australia
Kyumin Han and Joon Hyuk Lim	Samsung Bioepis, 107 Cheomdan-Daero, Yeonsu-gu, Incheon, 406-840	Republic of Korea
Manuel Navarro and Daniela Lorenzo	mAbxience SAU, Carlos Villate 5148, Munro, Buenos Aires, 1605	Argentina
Pankaj Kalita and Sanjay Bandyopadhyay	Zydus, Cadila Healthcare Ltd., Zydus Research Centre, Sarkhej Bavla N.H. No. 8A., Moraiya, Ahmedabad, 382213	India
Parvathy Harikumar and Haiyan Jia	Cytokines and Growth Factors Section, Biotherapeutics Group, NIBSC, Blanche Lane, South Mimms, Potters Bar, Herts, EN6 3QG	UK
Shubrata Khedkar and Mitali Samaddar	United States Pharmacopeia–India (P) Ltd., Plot D6 and D8, IKP Knowledge Park, Genome valley, Shameerpet, R.R. Dist. Telangana, Hyderabad, 500078	India
Nripendra Nath Mishra, Subhash Chand, Ratnesh K. Sharma and J. P. Prasad	National Institute of Biologicals, A-32, Sector-62, Noida, 201309	India
Tina Kneeland and David Cirelli	Pfizer, Analytical Research and Development, 1 Burtt Rd, Andover, Massachusetts 01810	USA
Valérie Ridoux and Jean-Claude Ourlin	ANSM, 635 rue de la garenne, Vendargues, 34740	France
Yangdong Sun	Innoventbio, 168 Dongping Street, Industrial Park, Suzhou, 215123	China
Yingchun LI and Tongjie Xu	CTTQ Pharma, No. 1099 Fuying Road, Jiangning Dist, Nanjing, 211100	China
Yujie Zhang	Teruisi Pharmaceutical Inc., 3rd Floor, Building 5, 1366 Hongfeng Road, South Lake Tai Scientific Innovation Center, Huzhou, 313000	China

**Table 3:** Mean fill parameters of final lyophilized Bevacizumab preparations.

Ampoule Code	Study Code	Protein (Predicted µg)	Mean Fill Weight (ng)	CV Fill Weight (%)	Mean Residual Moisture (%)	CV Residual Moisture (%)	Mean Headspace Oxygen (%)	CV Headspace Oxygen (%)
18/210	A, C	~53	1.0083 (241)	0.2193	0.56031 (12)	13.59	0.15 (12)	40.5
18/214	Β	~50	1.0100 (33)	0.2187	0.08349 (12)	11.21	0.34 (12)	32.2
18/216	D	~43	1.0093 (10)	0.1450	0.11034 (6)	8.49	0.35 (6)	15.5

The numbers in parentheses indicate the number of determinations. CV: Coefficient of variance.

**Table 4:** Summary of VEGF165 neutralisation assays contributing to the study.

Bioassay Type	Cell Line	Number of Participants	VEGF165 (U/mL) ^a^	Assay Period (Hours)	Assay Readout	Readout Reagent ^b^
Anti-proliferation	HUVEC	13	10–50	48–99	Absorbance (3)	CCK-8
					Fluorescence (8)	alamarBlue (6), CellTiter-Blue (1), resazurin dye (1)
					Luminescence (2)	CellTiter-Glo^®^
Reporter gene	HEK293	12	3.75–75	3.5–18	Luminescence	Bio-Glo™ luciferase (6), Bright-Glo™ luciferase (4), Steady-Glo^®^ luciferase (1)
Enzyme-fragment complementation	HEK293	2	11–12	16–20	Luminescence	PathHunter^®^ bioassay detection kit

^a^ Laboratory 5 used a commercial VEGF165 at 100 ng/mL. ^b^ Laboratory 16 provided readout units of luminescence and absorbance but not any information on readout reagents. The numbers in parentheses indicate the number of participants.

**Table 5:** Summary of binding assays employed in the collaborative study.

Assay Type	Number of Participants	IH Standard	Assay Description	Detection Reagent	Assay Readout	Readout Reagent
ELISA	13	Yes (or Avastin)	Bevacizumab binds to VEGF165 coated plate	Goat anti Human IgG-HRP	Absorbance	TMB substrate
Competitive binding	1	Yes	Bevacizumab and VEGF165 complex is added to capture plate	Anti-biotinylated VEGF	Absorbance	SureBlue™ TMB substrate
Biolayer interferometry	1	Avastin	Bevacizumab binds to biotinylated VEGF165 captured onto streptavidin biosensor	Not relevant	Response binding rate (nm/s)	Not relevant

Nine laboratories had in-house (IH) manufactured reference standards (expression host: Chinese hamster ovary cells), two laboratories used the drug product Avastin^®^ (Roche) and one laboratory had no IH reference standard.

**Table 6:** Individual laboratory geometric mean relative potency estimates for HUVEC assays.

Lab Code	Potency Relative to Candidate (Sample A)						Potency Relative to In-House Reference					
	Sample B			Sample C			Sample A			Sample B		
	GM	GCV	N	GM	GCV	N	GM	GCV	N	GM	GCV	N
04	1.19	59.69	5	1.01	124.2	7	1.08	61.61	9	1.32	144.8	4
05	0.78	15.51	9	0.98	16.29	9	0.87	12.25	12	0.67	14.47	9
06	0.80	7.98	8	0.96	7.20	8	1.10	9.07	9	0.89	8.49	8
07a ^a^	0.79	5.66	8	1.01	5.05	8	1.02	7.51	7	0.79	7.14	9
07b ^a^	0.75	17.29	3	1.01	5.46	4	1.08	n/a	1	0.81	9.65	4
08	0.98	24.38	12	1.13	47.25	11	1.20	32.77	14	1.13	42.28	12
09	0.86	16.02	11	1.02	10.31	10	-	-	-	-	-	-
12	0.99	18.31	6	1.04	55.68	4	-	-	-	-	-	-
14	0.98	26.27	7	0.96	40.82	8	1.59	12.26	7	1.54	26.14	5
21	0.93	10.62	8	0.99	14.39	8	1.04	8.08	12	0.97	8.62	8
22	1.03	33.38	8	1.08	18.93	9	-	-	-	-	-	-
23	0.81	3.98	12	1.01	5.47	12	2.03	8.82	12	1.64	9.32	12
25a	0.70	31.26	5	0.93	3.22	6	0.95	7.14	9	0.67	42.71	5
25b	0.66	n/a	1	0.77	3.17	6	1.26	0.82	3	n/a	n/a	n/a

^a^ Potencies calculated using a parallel line model. GM: Geometric Mean, GCV: Intra-laboratory Geometric Coefficient of Variation (%) and not calculated if N < 3, N: Number of valid estimates, n/a: Not calculated or no valid estimates obtained, -: Not calculated due to the lack of in-house reference standards.

**Table 7:** Individual laboratory geometric mean relative potency estimates for RGA/EFC assays.

Assay Type	Lab Code	Potency Relative to Candidate (Sample A)						Potency Relative to In-House Reference					
		Sample B			Sample C			Sample A			Sample B		
		GM	GCV	N	GM	GCV	N	GM	GCV	N	GM	GCV	N
RGA	01	0.89	11.86	11	1.04	10.93	8	1.40	n/a	1	1.42	n/a	1
RGA	02	0.77	20.85	9	0.91	19.84	9	1.14	9.78	9	0.88	17.53	9
RGA	04	0.86	10.00	9	1.02	10.30	9	1.04	8.89	12	0.88	6.70	9
RGA	05	0.80	7.14	9	1.01	7.98	9	1.01	13.48	12	0.86	12.46	9
RGA	08	0.95	12.66	6	0.90	n/a	2	1.38	14.85	5	1.26	22.26	5
RGA	10	0.83	9.84	9	1.06	16.08	9	1.06	13.30	9	0.85	16.52	7
RGA	13	0.74	29.12	8	0.94	52.00	7	1.22	28.29	6	1.01	27.61	6
RGA	15	0.98	30.94	3	0.90	n/a	2	1.26	35.18	3	1.25	32.38	3
RGA	16	0.73	n/a	1	-	-	-	0.95	n/a	1	1.04	n/a	1
RGA	17	0.89	8.92	9	1.03	7.41	8	1.01	11.31	12	0.87	6.88	9
RGA	18	0.82	7.39	6	0.98	9.54	6	n/a	n/a	n/a	n/a	n/a	n/a
RGA	19	0.85	5.74	9	1.01	10.15	8	1.17	6.83	9	0.99	5.76	9
EFC	21	0.92	8.81	7	0.87	12.58	7	1.17	17.21	10	1.14	26.03	7
EFC	24	0.73	6.58	9	0.94	18.94	9	-	-	-	-	-	-

RGA: reporter gene assay, EFC: enzyme-fragment complementation, GM: Geometric Mean, GCV: Intra-laboratory Geometric Coefficient of Variation (%) and not calculated if N < 3, N: Number of valid estimates, n/a: Not calculated or no valid estimates obtained, -: Not calculated due to the lack of in-house reference standards.

**Table 8:** Individual laboratory geometric mean relative potency estimates for binding assays.

Lab Code	Potency Relative to Candidate (Sample A)						Potency Relative to In-House Reference					
	Sample B			Sample C			Sample A			Sample B		
	GM	GCV	N	GM	GCV	N	GM	GCV	N	GM	GCV	N
02	0.88	6.07	9	1.01	9.39	9	1.01	6.38	9	0.90	6.73	9
03 ^a^	0.90	2.73	4	1.00	2.98	4	1.24	4.92	4	1.11	6.96	4
04	0.99	11.02	9	1.15	16.42	9	1.01	15.73	12	0.97	17.03	9
05	0.88	7.02	9	0.99	12.83	9	0.96	10.35	9	0.84	12.36	9
10	0.86	6.05	9	0.99	6.02	9	1.00	7.41	9	0.86	9.24	9
11	0.90	n/a	1	0.81	n/a	1	-	-	-	-	-	-
13	0.90	9.33	9	1.06	14.60	9	1.02	13.19	9	0.93	21.33	9
15	0.77	27.87	3	1.06	19.44	3	1.04	21.95	3	0.80	23.46	3
16	0.92	n/a	1	0.76	n/a	1	1.01	5.17	3	0.98	n/a	2
18	0.92	11.72	9	1.02	2.62	9	1.10	4.39	9	1.01	11.27	9
19	0.85	10.24	8	0.96	19.15	8	2.85	20.75	8	2.44	29.40	8
20	0.86	6.96	9	0.97	4.66	9	1.10	8.08	11	0.97	14.93	8
21	0.92	4.81	9	1.05	4.26	9	1.04	4.91	9	0.96	6.11	9
23	0.83	2.10	9	0.99	4.28	9	1.14	7.09	9	0.95	7.26	9

^a^ Potencies calculated using a parallel line model. GM: Geometric Mean, GCV: Intra-laboratory Geometric Coefficient of Variation (%) and not calculated if N < 3, N: Number of valid estimates, n/a: Not calculated or no valid estimates obtained, -: Not calculated due to the lack of in-house reference standards.

**Table 9:** Overall geometric mean relative potency estimates for VEGF165 neutralising and binding assays.

Method	Sample	Potencies Relative to Sample A					Potencies Relative to IH Reference				
		GM	LCL	UCL	GCV	N	GM	LCL	UCL	GCV	N
Neutralisation (All) ^a^	A	-	-	-	-	-	1.16	1.05	1.28	21.87	18
	B	0.85	0.82	0.89	11.35	25	0.98	0.87	1.10	27.98	19
	C	1.00	0.97	1.02	5.90	23	1.14	1.03	1.26	21.94	18
	D	0.86	0.81	0.91	12.59	17	0.95	0.80	1.13	30.29	12
Neutralisation (HUVEC) ^a^	A	-	-	-	-	-	1.18	0.93	1.50	32.91	8
	B	0.86	0.79	0.93	13.61	12	0.96	0.75	1.24	39.14	9
	C	1.01	0.98	1.04	5.39	12	1.16	0.94	1.42	30.90	9
	D	0.84	0.79	0.90	9.86	10	0.95	0.72	1.25	38.60	8
Neutralisation (RGA/EFC) ^a^	A	-	-	-	-	-	1.14	1.06	1.23	10.73	10
	B	0.85	0.80	0.89	9.38	13	0.99	0.88	1.10	16.89	10
	C	0.98	0.94	1.02	6.37	11	1.13	1.04	1.22	10.96	9
	D	0.88	0.77	1.01	16.22	7	0.96	0.83	1.10	9.37	4
Binding ^b^	A	-	-	-	-	-	1.06	1.01	1.11	7.50	11
	B	0.88	0.85	0.91	6.26	12	0.93	0.88	0.99	9.61	11
	C	1.02	0.99	1.05	5.08	12	1.09	1.03	1.14	7.75	11
	D	0.81	0.78	0.84	5.16	8	0.86	0.79	0.95	11.80	8

^a^ Lab 04 HUVEC results excluded, Lab 25b results excluded, and all lab GM potencies with N < 3 excluded. ^b^ Lab 11 and 16 excluded and Lab 19 excluded relative to in-house reference. IH: in-house, RGA: reporter gene assay, EFC: enzyme-fragment complementation, GM: Geometric mean, LCL: Lower 95% confidence limit, UCL: Upper 95% Confidence limit, GCV: Inter-laboratory geometric coefficient of variation (%), N: Number of laboratories.

**Table 10:** Summary of EC_50_ estimates (ng) ^a^ for selected HUVEC-based neutralization assays using a fixed amount of VEGF (25 Units).

Sample	GM	LCL	UCL	GCV	N
A	132.2	63.9	273.6	99.9	6
B	152.8	78.2	298.5	89.3	6
C	141.5	61.5	325.5	121.2	6
IH	194.6	117.6	322.1	50.1	5

^a^ EC_50_ estimates (ng) calculated based on the assumed protein content of 50 µg for the samples. GM: Geometric mean, LCL: Lower 95% confidence limit, UCL: Upper 95% Confidence limit, GCV: Inter-laboratory geometric coefficient of variation (%), N: Number of laboratories.

## Data Availability

Not applicable.

## Supplementary Materials

The following are available online at https://www.mdpi.com/article/10.3390/biom11111610/s1, Figure S1: Laboratory geometric mean EC_50_ estimates for samples A and C versus final VEGF concentrations (U/mL) in neutralizations assays. Table S1: Brief details of VEGF neutralisation assays contributed to the study. Table S2: Brief details of VEGF binding assays contributed to the study. Table S3: Summary of results from accelerated temperature degradation studies of candidate preparation 18/210 tested using the HUVEC-based VEGF neutralization assays. Table S4: Summary of results from reconstitution stability studies of candidate preparation 18/210 tested using the HUVEC-based VEGF neutralization assays. Table S5: Summary of results from freeze–thaw stability studies of candidate preparation 18/210 tested using the HUVEC-based VEGF neutralization assays.
