# Supplementary material for: The First WHO International Standard for Harmonizing the Biological Activity of Bevacizumab
Source: Biomolecules. 2021 Oct 30;11(11):1610. doi: 10.3390/biom11111610 (PMC8615914; doi:10.3390/biom11111610)
Supplement: Supplementary file 1 [file biomolecules-11-01610-s001.zip › biomolecules-1393592-supplementary.pdf]

biomolecules

MDPI

Supplementary Materials

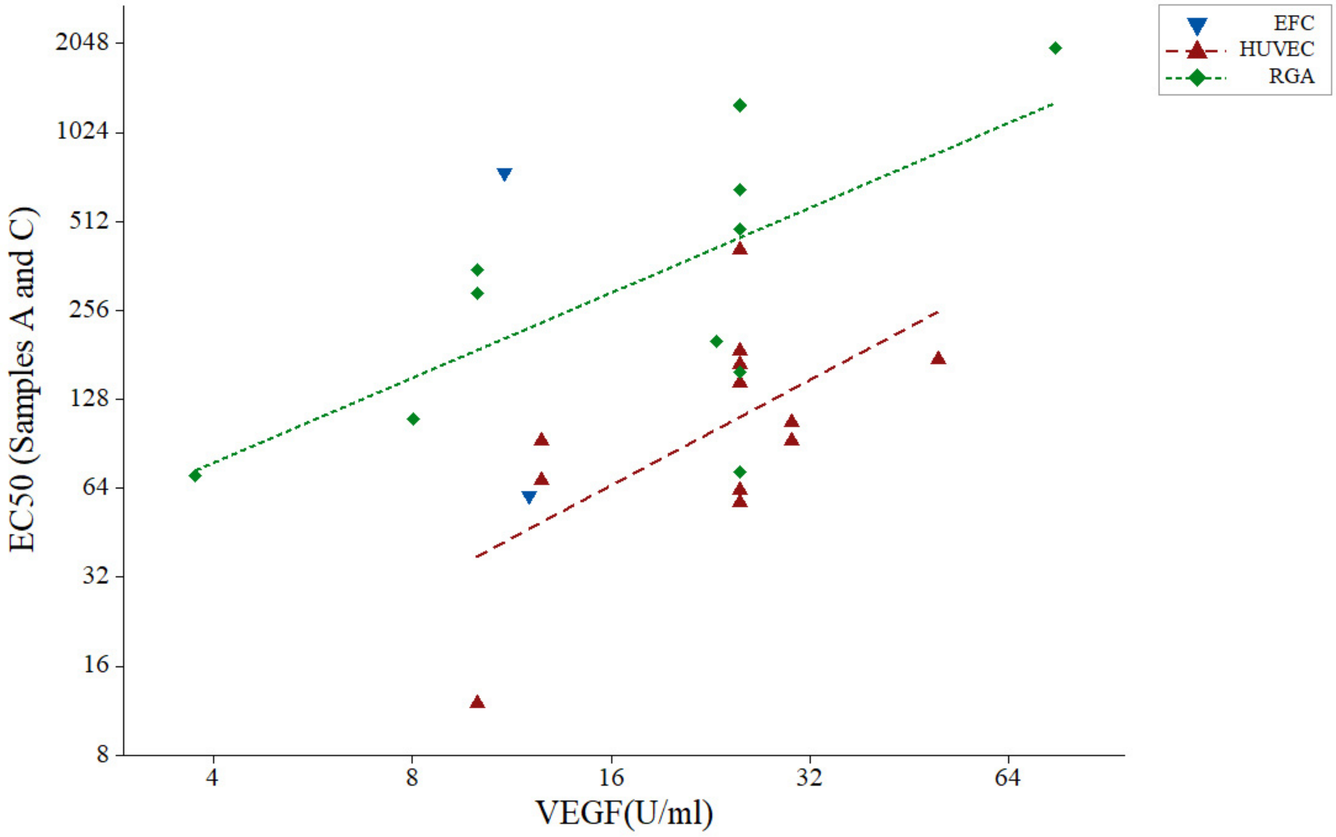

Figure S1. Laboratory geometric mean EC_{50} estimates for samples A and C versus final VEGF concentrations (U/ml) in neutralizations assays.

Table S1. Brief details of VEGF neutralisation assays contributed to the study.

Lab Code	Cell Line	Assay Type	Final VEGF Concentration (U/mL)	In-house Standard	Incubation Time (hrs)	Assay Readout	Readout Reagent
4	HUVEC	Anti- proliferation	25	MIH	66–72	Absorbance	CCK-8
5	HUVEC	Anti- proliferation	100 ng/mL[a]	MIH	89.5	Absorbance	CCK-8
6	HUVEC	Anti- proliferation	25	MIH	68–72	Luminescence	CellTiter-Glo®
7	HUVEC	Anti- proliferation	40	MIH	90-98	Fluorescence	alamarBlue™
8	HUVEC	Anti- proliferation	25	A	~ 72	Fluorescence	alamarBlue™
9	HUVEC	Anti- proliferation	25	Not available	99	Fluorescence	alamarBlue™
11	HUVEC	Anti- proliferation	12.5	Not available	72-80	Fluorescence	alamarBlue™
12	HUVEC	Anti- proliferation	10	Not available	65-70	Fluorescence	alamarBlue™
14	HUVEC	Anti- proliferation	25	R	72	Fluorescence	Resazurin dye
21	HUVEC	Anti- proliferation	30	MIH	93-99	Absorbance	CCK-8
22	HUVEC	Anti- proliferation	12.5	Not available	3 ± 30 min	Fluorescence	alamarBlue™
23	HUVEC	Anti- proliferation	25	MIH	48	Luminescence	CellTiter-Glo®
25	HUVEC	Anti- proliferation	30 and 50	A	96	Fluorescence	CellTiter-Blue
1	HEK293/VEGFR2 (iLite assay-ready cells)	Reporter gene	25	A	18	Luminescence	Bright-Glo™ luciferase
2	HEK293-KDR-NFAT-RE-Luc2P	Reporter gene	25	MIH	5–6	Luminescence	Bright-Glo™ luciferase
4	HEK293-KDR-NFAT-RE-Luc2P	Reporter gene	23	MIH	5–6	Luminescence	Bio-Glo™ luciferase

5	HEK293-KDR-Luc2P	Reporter gene	25	MIH	18	Luminescence	Bio-Glo™ luciferase
8	HEK293/VEGFR2 (iLite assay-ready cells)	Reporter gene	25	A	18	Luminescence	Bright-Glo™ luciferase
10	HEK293-KDR-NFAT-RE-Luc2P	Reporter gene	25	A	6	Luminescence	Bright-Glo™ luciferase
13	HEK293-KDR-NFAT-RE-Luc2P	Reporter gene	3.75	MIH	6	Luminescence	Bio-Glo™ luciferase
15	HEK293-KDR-NFAT-RE-Luc2P	Reporter gene	25	MIH	6	Luminescence	Bio-Glo™ luciferase
16	HEK293-VEGFRR2-NFAT-Luciferase	Reporter gene	8	A	4	Luminescence	Not available
17	HEK293-KDR-NFAT-RE-Luc2P	Reporter gene	10	A	6	Luminescence	Bio-Glo™ luciferase
18	HEK293-VEGFR2-NFAT-Luc	Reporter gene	75	MIH	3.5–6	Luminescence	Steady-Glo® luciferase
19	HEK293T-VEGFR2-NFAT-Luciferase	Reporter gene	10	MIH	4–6	Fluorescence	Bio-Glo™ luciferase
21	HEK293-KDR/KDR	Enzyme- fragment complementation	12	MIH	16–20	Luminescence	PathHunter® bioassay detection kit
24	HEK293-KDR/KDR	Enzyme- fragment complementation	11	Not available	16–18	Luminescence	PathHunter® bioassay detection kit

MIH: Manufactured in house (expression host: Chinese hamster ovary cells; form: IgG1); A: Avastin® Roche drug product (expression host: Chinese hamster ovary cells; form: IgG1); R: Anti-human VEGF antibody (research grade Bevacizumab biosimilar; R&D Systems). [a] R&D Systems (catalog number: 293-VE-050).

Table S2. Brief details of VEGF binding assays contributed to the study.

Lab Code	Assay Type	In-house Standard	Assay Description	Detection Reagent	Assay Readout	Readout Reagent
2	ELISA	MIH	Bevacizumab binds to VEGF165 coated plate	Goat anti Human kappa-HRP	Absorbance	TMB substrate
3	Biolayer interferometry	A	Bevacizumab binds to biotinylated VEGF165 captured onto streptavidin biosensor	Not available	Response binding rate (nm/s)	Not available
4	ELISA	MIH	Bevacizumab binds to VEGF165 coated plate	Goat anti-Human IgG-HRP	Absorbance	TMB substrate
5	ELISA	MIH	Bevacizumab binds to VEGF165 coated plate	Goat anti-Human IgG-Fc Fragment Specific	Absorbance	TMB substrate
10	ELISA	A	Bevacizumab binds to VEGF165 coated plate	Goat anti-Human IgG Fc-HRP	Absorbance	TMB substrate
11	ELISA	Not available	Bevacizumab binds to VEGF165 coated plate	Goat anti-human kappa-HRP	Absorbance	TMB substrate
13	ELISA	MIH	Bevacizumab binds to VEGF165 coated plate	Goat anti-human IgG Fc-HRP	Absorbance	TMB substrate
15	ELISA	MIH	Bevacizumab binds to VEGF165 coated plate	Goat anti-human IgG Fc-HRP	Absorbance	TMB substrate
16	ELISA	A	Not available	Not available	Absorbance	Not available
18	ELISA	MIH	Bevacizumab binds to VEGF165 coated plate	anti-human IgG-HRP	Absorbance	TMB substrate
19	ELISA	MIH	Bevacizumab binds to VEGF165 coated plate	Goat anti-Human IgG Fc-HRP	Absorbance	TMB substrate
20	Competitive binding	MIH	Bevacizumab/VEGF165 complex is added to capture plate	Anti-rhVEGF-biotinylated	Absorbance	SureBlue™ TMB substrate
21	ELISA	MIH	Bevacizumab binds to VEGF165 coated plate	Goat anti-Human IgG-HRP	Absorbance	TMB substrate
23	ELISA	MIH	Bevacizumab binds to VEGF165 coated plate	Goat anti-Human IgG-HRP	Absorbance	TMB substrate

MIH: Manufactured in house (expression host: Chinese hamster ovary cells; form: IgG1); A: Avastin® Roche drug product (expression host: Chinese hamster ovary cells; form: IgG1).

Table S3. Summary of results from accelerated temperature degradation studies of candidate preparation 18/210 tested using the HUVEC-based VEGF neutralization assays.

Time Stored (years)	Storage Temperature (°C)	LCL	Relative Potency to -70°C	UCL
0.833	−20	0.95	1.07	1.19
0.833	+4	0.34	0.94	2.61
0.833	+20	0.95	1.03	1.12
0.833	+37	0.94	1.00	1.07
0.833	+45	0.89	0.96	1.04
0.917	−20	0.69	1.00	1.43
0.917	+4	0.84	0.92	1.01
0.917	+20	0.81	0.95	1.12
0.917	+37	0.73	1.00	1.35
0.917	+45	0.78	0.98	1.23

Geometric mean potency derived from 6 estimates for -20°C, 3 estimates for +4°C, and 9 estimates for all other cases. LCL and UCL: Lower and Upper 95% confidence limits.

Table S4. Summary of results from reconstitution stability studies of candidate preparation 18/210 tested using the HUVEC-based VEGF neutralization assays.

Temperature (°C)	Time (Days)	95% Lower Confidence Limit	Relative Potency to a Freshly Reconstituted Ampoule	95% Upper Confidence Limit
+4	1	0.89	1.07	1.29
+4	7	0.87	1.23	1.73
Room temperature	1	1.04	1.11	1.19
Room temperature	7	0.93	1.13	1.37

Geometric mean potency derived from 5 estimates for 1 day at +4°C and 4 estimates for all other cases. LCL and UCL: Lower and Upper 95% confidence limits.

Table S5. Summary of results from freeze-thaw stability studies of candidate preparation 18/210 tested using the HUVEC-based VEGF neutralization assays.

Number of freeze/thaw cycles	LCL	Relative potency	UCL
1	0.87	1.08	1.34
2	0.95	1.15	1.39
3	0.75	1.05	1.48
4	0.86	1.00	1.17

Geometric mean potency derived from 6 estimates for all cases. L and UCL: Lower and Upper 95% confidence limits.uthors, add author initials).
